# Qualitative evaluation of artificial intelligence-generated weight management diet plans

**DOI:** 10.3389/fnut.2024.1374834

**Published:** 2024-03-21

**Authors:** Dong Wook Kim, Ji Seok Park, Kavita Sharma, Amanda Velazquez, Lu Li, John W. Ostrominski, Tram Tran, Robert H. Seitter Peréz, Jeong-Hun Shin

**Affiliations:** ^1^Division of Endocrinology, Diabetes and Hypertension, Center for Weight Management and Wellness, Brigham and Women's Hospital, Boston, MA, United States; ^2^Department of Medicine, Section of Endocrinology, Diabetes, Nutrition & Weight Management, Boston University Chobanian & Avedisian School of Medicine, Boston, MA, United States; ^3^Department of Gastroenterology, Hepatology & Nutrition, Cleveland Clinic, Cleveland, OH, United States; ^4^Department of Medicine, Weight Management and Metabolic Health Center, Cedars Sinai Hospital, Los Angeles, CA, United States; ^5^Division of Cardiovascular Medicine, Brigham and Women’s Hospital and Harvard Medical School, Boston, MA, United States; ^6^Department of Internal Medicine, Hanyang University College of Medicine, Seoul, Republic of Korea

**Keywords:** AI, diet plan, weight management, ChatGPT, dietary intervention

## Abstract

**Importance:**

The transformative potential of artificial intelligence (AI), particularly via large language models, is increasingly being manifested in healthcare. Dietary interventions are foundational to weight management efforts, but whether AI techniques are presently capable of generating clinically applicable diet plans has not been evaluated.

**Objective:**

Our study sought to evaluate the potential of personalized AI-generated weight-loss diet plans for clinical applications by employing a survey-based assessment conducted by experts in the fields of obesity medicine and clinical nutrition.

**Design, setting, and participants:**

We utilized ChatGPT (4.0) to create weight-loss diet plans and selected two control diet plans from tertiary medical centers for comparison. Dietitians, physicians, and nurse practitioners specializing in obesity medicine or nutrition were invited to provide feedback on the AI-generated plans. Each plan was assessed blindly based on its effectiveness, balanced-ness, comprehensiveness, flexibility, and applicability. Personalized plans for hypothetical patients with specific health conditions were also evaluated.

**Main outcomes and measures:**

The primary outcomes measured included the indistinguishability of the AI diet plan from human-created plans, and the potential of personalized AI-generated diet plans for real-world clinical applications.

**Results:**

Of 95 participants, 67 completed the survey and were included in the final analysis. No significant differences were found among the three weight-loss diet plans in any evaluation category. Among the 14 experts who believed that they could identify the AI plan, only five did so correctly. In an evaluation involving 57 experts, the AI-generated personalized weight-loss diet plan was assessed, with scores above neutral for all evaluation variables. Several limitations, of the AI-generated plans were highlighted, including conflicting dietary considerations, lack of affordability, and insufficient specificity in recommendations, such as exact portion sizes. These limitations suggest that refining inputs could enhance the quality and applicability of AI-generated diet plans.

**Conclusion:**

Despite certain limitations, our study highlights the potential of AI-generated diet plans for clinical applications. AI-generated dietary plans were frequently indistinguishable from diet plans widely used at major tertiary medical centers. Although further refinement and prospective studies are needed, these findings illustrate the potential of AI in advancing personalized weight-centric care.

## Introduction

Artificial intelligence (AI) holds transformative potential in the healthcare landscape (1). Its ability to enhance personalization in care delivery models is becoming increasingly evident (2). Weight-loss diet plans, considered crucial educational materials, are frequently provided in weight management clinics. High-quality personalized resources play a pivotal role in influencing clinical outcomes by ensuring that patients receive guidance tailored to their unique needs (3, 4). However, in the absence of AI assistance, the creation and implementation of personalized diet plans in real-world scenarios is a demanding task. This requires an intricate integration of diverse clinical and cultural variables, thereby presenting numerous challenges.

In this context, since the public release of the Chatbot Generative Pre-trained Transformer (ChatGPT) in November 2022, AI’s application in healthcare has become a noteworthy topic of discussion, attracting a surge in related academic publications (5). ChatGPT, an advanced language model, is capable of generating human-like text, thus facilitating engaging conversations, answering queries, and providing detailed information on a myriad of topics, including medicine and healthcare. Despite not being specifically designed for healthcare applications, ChatGPT can integrate data from multiple clinical sources and generate new outputs based on this combined information (6). This feature makes it an invaluable tool for creating personalized diet plans, demonstrating the substantial impact of AI in healthcare.

The objective of our study was to perform a qualitative evaluation of diet plans created by AI. We hypothesized that AI-generated diet plans are as practicable as currently employed diet plans and that AI has the capacity to generate personalized diet plans. In our investigation, we examined a general diet plan produced by ChatGPT by contrasting it with two control weight-loss diet plans that are currently utilized and offered to patients in weight management clinics. Moreover, we evaluated the ChatGPT-generated personalized diet plans for hypothetical patients with multiple complex medical conditions and distinct food preferences.

## Methods

The data collection phase of our study was conducted from April 27th, 2023 to May 8th, 2023 through an individual invitation-based survey. Additionally, an open-ended survey using an invitation link was conducted from April 15th, 2023 to May 8th, 2023. Survey participants included professionals specializing in obesity medicine or nutrition, such as dietitians, physicians, and nurse practitioners. We compiled a list of specialists from the American Board of Obesity Society and National Board of Physician Nutrition Specialists. This process resulted in 414 specialists with publicly available email addresses who were invited to participate in the survey via email. We also contacted 11 institutions known for their obesity or nutritional expertise and shared a survey link with these institutions. We managed all study data using Research Electronic Data Capture (REDCap) software, with the server located at Boston University Medical Center. Our study was reviewed by the Boston University Medical Campus and Boston Medical Center Institutional Review Board (IRB) and granted an exemption from the formal IRB process.

Initially, we selected two weight-loss diet plans from tertiary medical center clinics to serve as control diet plans for comparison with AI-generated plans. These plans are currently used as patient educational materials in hospitals’ weight management clinics. To ensure uniformity among the diet plans, we modified their format by altering the display order, removing illustrations, and converting the text format to unformatted text while keeping the content intact. Each plan was divided into three parts: general diet instructions, food choices that promote weight loss, and a five-day sample menu.

The ChatGPT model (version 4.0) was used to generate AI-based diet plans on March 16th, 2023. We refreshed the model before the first input and provided three key sentences consecutively, without any refreshing between the inputs. The outputs were converted into plain text to match the format of the control diet. The key sentences input to create the non-personalized diet plan were: “Create a diet instruction for weight loss,” “Make a list of foods to choose and avoid for weight loss,” and “Create a diet sample menu for five days” (Supplementary material 1). Furthermore, a personalized diet plan was developed for hypothetical patients with multiple health conditions. The patient had a history of stroke, chronic renal insufficiency with an estimated glomerular filtration rate (eGFR) of 52 mL/min/1.73 m^2^, severe acid reflux disease, shellfish allergy, and a preference for Spanish cuisine. We used an approach similar to that used for the standard diet plan, but with specifics adjusted for the patient’s health profile and preferences (Supplementary material 2). The AI-generated diet plans omitted greetings and warning expressions typically provided by ChatGPT.

The method employed in our study involved a three-part survey designed to gather expert feedback on diet plans generated by AI. In the first part of the survey, we collected demographic data, focusing on the respondents’ professional titles, years of experience, and how frequently they incorporated diet plans and sample menus in consultation with their patients. The second part of the survey included a thorough assessment of a general weight-loss diet plan created by ChatGPT. This was compared with control diet plans sourced from two different institutions. Participants were asked to review these plans and rate them on a series of variables, including effectiveness, which refers to a diet plan’s potential to cause a calorie deficit leading to weight loss; balanced-ness, which is a measure of the nutritional balance of the diet plan in macro-and micronutrient terms; comprehensiveness, an indication of the diet plan’s level of detail and its ease of use; flexibility, a measure of the variety of food choices from all the major food groups provided by the plan; applicability, an assessment of how readily a diet plan could be incorporated into a weight management program with minimal changes; and finally, an overall impression, which sought to gauge whether the respondent would recommend the diet to patients seeking to lose weight. A 0–10 Likert scale was used for this evaluation. In this section, we also asked experts to identify which diet plan was generated by AI and to provide their rationale for this selection. The third and final part of the survey was designed to assess personalized diet plans. It involved scoring the effectiveness and safety in terms of the diet plan’s suitability for a patient with specific medical conditions, applicability, and respondents’ likelihood of using the presented personalized diet plan. This evaluation also used a 0 to 10 Likert scale. To conclude the survey, we provided a free-text feedback form inviting experts to share their insights and thoughts on the personalized diet plan generated by AI.

Continuous variables are presented as means and standard deviations. However, for continuous variables that did not follow a normal distribution, the median and interquartile range are presented. Categorical variables were presented as frequencies and percentages. ANOVA was used to compare the three diet plans regarding effectiveness, balancedness, comprehensiveness, flexibility, applicability, and overall impression. All statistical analyses were conducted using statistical software R-4.2.3 (R Core Team, R Foundation for Statistical Computing, Vienna, Austria) and RStudio-2023.03.0 + 386 (RStudio Team, RStudio Inc., Boston, MA, United States). All reported *p*-values are 2-sided, with a value of <0.05 considered significant.

## Results

Ninety-five experts participated in our survey, but 28 were excluded from the analysis because of incomplete responses. Among the remaining 67 participants, 27 (40.3%) were physicians, 37 (55.2%) registered dietitians, and 3 (4.5%) nurse practitioners. All respondents specialized in obesity medicine or clinical nutrition, with a median professional experience of nine years.

**Figure 1:**
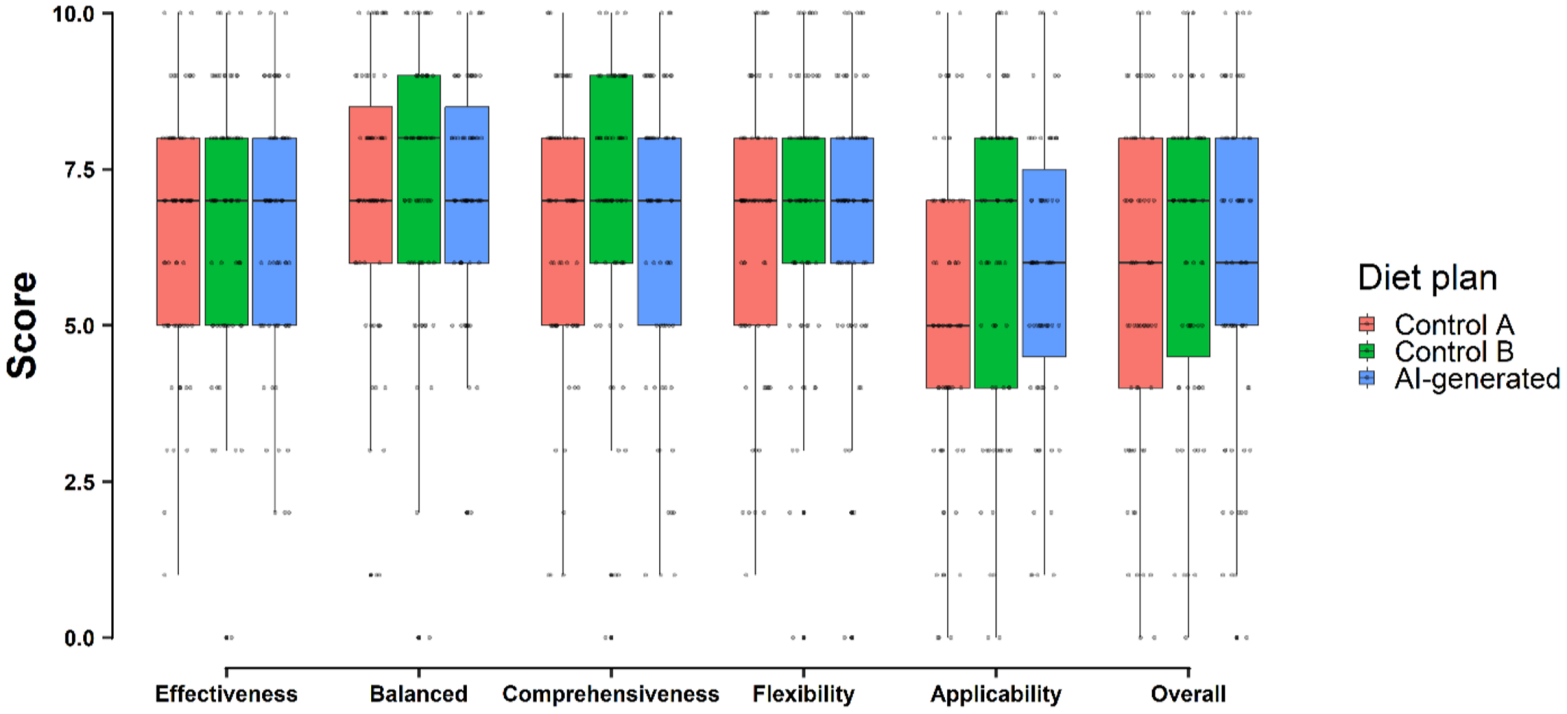
Comparison of the AI-generated diet plan with the diet plans currently used in two medical institutions.

The 67 respondents who completed the weight loss diet plan comparison did not show significant differences across the three diet plans in any evaluation category, including in the overall evaluation score. Across key domains, the AI-generated diet plan performed similarly as compared with the two control diet plans (Figure 1). Of the 67 experts, 53 (79.1%) reported being unable to distinguish which diet plan was AI-generated. Of the 14 survey respondents who stated they were able to identify the AI-generated plan, only 5 did so correctly. Interestingly, of the 53 participants who claimed to be unable to recognize the AI-generated plan, 24 chose it unknowingly when asked to identify which plan was AI-generated.

### Evaluating AI-generated personalized diet plans

**Figure 2:**
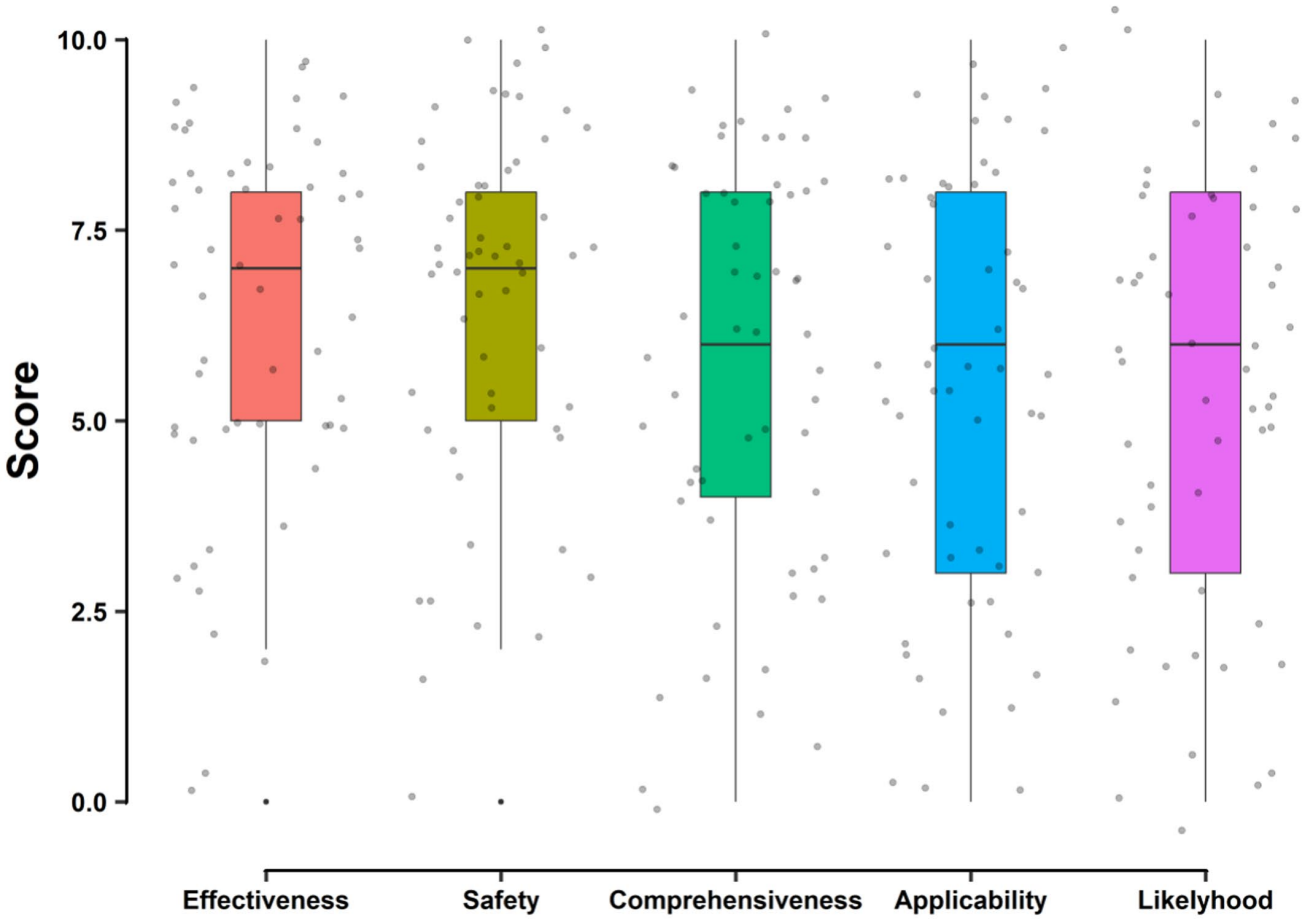
Qualitative evaluation of AI-generated personalized diet plans.

Of all survey participants, 57 completed the assessment of the AI-generated personalized diet plan. Among all the evaluation items, “Safety” received the highest mean score of 6.53, while “Intention to Use the Personalized Diet Plan” received the lowest mean score of 5.40 (Figure 2). A notable portion of the experts, 30 in total, provided comments about these diet plans. These observations are further examined in the Discussion section.

## Discussion

In response to the swift adoption of AI technology and the growing popularity of ChatGPT, this study delves into its potential applications in real medical scenarios, particularly in nutritional counseling. AI could theoretically enhance or even replace resources, such as UpToDate, providing clinicians with valuable tools to create differential diagnoses or preliminary treatment plans. However, concerns over inaccurate information and privacy breaches require ongoing and careful adjudication of these approaches (7). Nutritional counseling is actively practiced in hospitals. Although a significant amount of accumulated data and information is available, it is currently institution-specific and lacks standardization (8, 9). Therefore, in this study, we conducted research on the application and role of AI in this particular area. As there is no established diet plan, we compared it with diet plans currently utilized in clinical practice and evaluated its utility, safety, and feasibility through blind tests and expert opinions from professionals, such as physicians, registered dietitians, and nurse practitioners. After examining the study results, we found that AI-generated diet plans matched well with traditional clinical diet plans in all areas of assessment. This interesting discovery indicates the potential use of AI-generated diet plans in clinical settings.

Owing to individual variations in underlying health conditions and dietary preferences, a general diet plan cannot be universally applied to all patients. Therefore, we conducted this study to determine whether AI could help address these issues. Although the AI-generated personalized diet plan obtained slightly lower scores than the AI-generated non-personalized plan in areas such as effectiveness, comprehensiveness, and applicability, it still achieved mean evaluation scores that exceeded five. However, it is important to acknowledge that the AI-generated personalized diet plan was not assessed as a blind survey. As such, the evaluators’ awareness that the diet plan was AI-derived could have introduced bias, thereby affecting the scoring results (10).

Distinguishing AI-generated outputs from human writing, particularly those created by ChatGPT, presents a significant challenge (11). Our study reinforced this observation as only 5 out of 67 experts were able to accurately identify and select the AI-generated diet plan. These experts highlighted characteristics such as the broad comprehensiveness of the diet plan and the inclusion of atypical recommendations. Moreover, an intriguing finding emerged in which 24 experts who initially reported that they could not identify the AI-generated plan correctly selected the AI plan. Their reasoning revolved around nonspecific characteristics, such as the absence of brand names and meal preparations perceived as unrealistic. Therefore, although the task of identifying AI-generated diet plans is complex, some experts were able to pinpoint them, typically because of factors not directly related to the quality of the diet plan.

The experts’ commentary noted several limitations of the AI-generated personalized diet plan. A commonly addressed issue was the recommendation of ‘Tomato’ as a food item. This highlights a fundamental challenge for AI: navigating through complex and conflicting considerations. In our case, while tomatoes are integral to Spanish cuisine, they aren’t recommended for patients with severe gastroesophageal reflux disease (GERD) and advanced chronic kidney disease (CKD) due to their high electrolyte content (12). The AI may have prioritized the preference for Spanish cuisine over the restrictions related to CKD and GERD. These conflicting considerations were also evident in the differing views of experts regarding the protein content of the diet plan. One expert considered the excessive amount of protein in a patient with CKD, whereas another felt that it was insufficient for weight loss. Their contrasting viewpoints reflect the primary clinical condition on which each expert focused, whether it was weight loss or CKD (13). This reinforces that decision-making in real-world clinical scenarios often involves complex and conflicting factors, demonstrating the challenges that AI may face in producing optimal personalized diet plans (14). Furthermore, it emphasizes that AI-derived information may vary if there are no standard therapies in reality.

Further commentary from our experts highlighted several areas of potential improvement in AI-generated diet plans. A notable point of concern was the lack of precise portion sizes in personalized diet plans. Additionally, some experts have pointed out that the frequent recommendation of high-priced ingredients could pose affordability issues for patients. Another significant observation is the lack of recommendation specificity. For instance, the recommendation for “Spanish cuisine” was seen as overly broad, while suggesting a dish like “Spanish Moussaka” could be problematic due to its various interpretations. These issues may be the result of nonspecific or vague input data provided to ChatGPT. Therefore, refining the input parameters to enhance their specificity could help generate more accurate and practical outputs (15). Ultimately, this might improve the quality of the diet plans produced by AI, making them more applicable and beneficial for patients.

As outlined above, there are several limitations in applying an AI-generated personalized diet plan in a real clinical situation, specifically within the context of a weight management clinic. Despite these constraints, AI-generated diet plans received a mean score above neutral for all categories. Given the inherent complexity of generating such specialized diet plans, we consider this score promising. One expert highlighted the potential utility of these plans for individuals who require different languages. The output can be easily and continuously modified by adding specific conditional inputs such as changing the language or adjusting certain ingredients. The adaptability of the ChatGPT-created plan can help mitigate and improve some of the limitations discussed earlier (16).

Several crucial factors must be considered before applying AI-generated diet plans. First, the potential copyright issues should be addressed (17). As of now, content created by large language model-based AI does not infringe upon copyright laws because it does not merely memorize the context of the original content. However, this non-memorization aspect may be less prevalent in certain unique and specific situations, which could potentially give rise to copyright infringements (18). Another concern pertains to the potential generation of incorrect information using AI. This phenomenon, known as “hallucination,” occurs when the model produces a highly confident yet erroneous statement (19, 20). Current AI models, like ChatGPT, lack the capability to fact-check their outputs. Therefore, it remains the responsibility of human experts to validate these outputs (21). Consequently, any AI-created diet plan should undergo expert review before it is released to the public. Moreover, adherence to the Health Insurance Portability and Accountability Act provisions is critical when generating personalized diet plans (21, 22). Although ChatGPT cannot store or recall personal data, it is crucial to use generalized terms in the creation of personalized diet plans to avoid the use of any protected health information. Hence, it is vital to balance the power of AI personalization with privacy and legal compliance.

This study had several limitations. Primarily, the AI-generated diet plans exhibited a degree of inconsistency, likely owing to inherent randomness in the output generation of the AI chatbot model (23, 24). The model was engineered to produce a range of responses rather than consistently offering identical solutions. Thus, identical inputs can result in slightly varying diet plans, which could affect scoring outcomes. This inconsistency poses a challenge in the validation of AI-based diet plans. While adjusting the ChatGPT module’s settings can potentially reduce response diversity and minimize inconsistencies, these options are currently inaccessible to general users (24). Another constraint of this study is the lack of a universally recognized gold standard for weight-loss diet plans. Therefore, even if AI-created diet plans yield similar or superior scores compared to various institutional control diet plans, it does not conclusively affirm that the ChatGPT-created diet plan is suitable for real-world clinical scenarios. Finally, despite our efforts to categorize numerous evaluation aspects, the assessments remain inherently subjective. At present, no objective scoring system is available to evaluate diet plans, which further complicates the assessment and comparison processes.

## Conclusion

Our study highlights the potential of AI-generated diet plans for real-world clinical applications, despite certain limitations. The quality of these AI-generated plans paralleled that of the existing patient education materials, underlining their prospective utility. To the best of our knowledge, this is the first study in which multiple experts evaluate AI-created outcomes for potential clinical implications. Although our research primarily concentrated on qualitative evaluations by experts, additional validation of AI-generated diet plans is crucial for a more robust application of AI in diet planning. Future investigations should adopt a quantitative approach, involving the evaluation of diverse outputs from multiple AI tools across a broad spectrum of clinical conditions and scenarios.

## Data availability statement

The original contributions presented in the study are included in the article/Supplementary material, further inquiries can be directed to the corresponding authors.

## Author contributions

DK: Conceptualization, Investigation, Supervision, Validation, Writing – original draft, Writing – review & editing. JP: Data curation, Methodology, Writing – review & editing. KS: Data curation, Investigation, Methodology, Writing – review & editing. AV: Data curation, Formal analysis, Investigation, Validation, Writing – review & editing. LL: Data curation, Formal analysis, Investigation, Writing – review & editing. JO: Validation, Writing – review & editing. TT: Methodology, Writing – review & editing. RS: Data curation, Writing – review & editing. J-HS: Formal analysis, Writing – original draft, Writing – review & editing.
