# Supplementary material for: Qualitative evaluation of artificial intelligence-generated weight management diet plans
Source: Front Nutr. 2024 Mar 21;11:1374834. doi: 10.3389/fnut.2024.1374834 (PMC10991711; doi:10.3389/fnut.2024.1374834)
Supplement: Supplementary file 1 [file Data_Sheet_1.pdf]

Diet plan C

<Instruction>
Remember, weight loss is a gradual process, and consistency is key. Stick to your plan, make adjustments as needed, and be patient with yourself.

1. Set a realistic goal: Determine a healthy weight loss target, typically aiming to lose 1-2 pounds per week. Track your progress and adjust your diet as needed.
2. Calculate your daily calorie needs: Estimate your daily caloric needs using an online calculator that factors in your age, gender, weight, height, and activity level. To lose weight, subtract 500-1000 calories from your maintenance calories to create a calorie deficit.
3. Prioritize whole, nutrient-dense foods: Include a variety of fruits, vegetables, whole grains, lean proteins, and healthy fats in your diet. These foods will provide essential nutrients and help you feel full and satisfied.
4. Plan balanced meals: Aim for the following macronutrient distribution in each meal:
    - 45-60% carbohydrates (focus on whole grains and fiber-rich sources)
    - 20-35% fats (emphasize healthy fats from sources like avocados, nuts, and olive oil)
    - 10-35% proteins (choose lean sources like poultry, fish, beans, and tofu)
5. Control portion sizes: Use measuring cups, food scales, or visual cues to manage portion sizes and avoid overeating.
6. Stay hydrated: Drink at least 8 cups (64 ounces) of water per day to support overall health and weight loss. Limit sugary drinks and alcohol.
7. Limit added sugars and processed foods: Reduce consumption of refined carbohydrates, added sugars, and processed foods, which can contribute to weight gain.
8. Eat mindfully: Pay attention to your hunger cues and eat slowly, savoring each bite. Avoid eating when you're distracted, stressed, or emotional.
9. Plan snacks: Choose healthy, nutrient-dense snacks, such as fruits, vegetables, nuts, or yogurt, to help manage hunger between meals.
10. Allow for occasional treats: It's okay to indulge in your favorite treats occasionally, but be mindful of portion sizes and frequency.
11. Exercise regularly: Incorporate both aerobic exercise and strength training into your routine to help accelerate weight loss and improve overall health.
12. Get support: Share your weight loss journey with friends, family, or join a support group to stay motivated and accountable.

<Food to Choose>
Fruits:
Berries (strawberries, blueberries, raspberries, blackberries), Apples, Oranges, Pears, Kiwi, Grapefruit, Watermelon, Cherries, Pineapple, Papaya

Vegetables:
Leafy greens (spinach, kale, arugula, Swiss chard), Broccoli, Cauliflower, Bell peppers, Carrots, Zucchini, Cucumbers, Brussels sprouts, Asparagus, Eggplant

Whole grains:
Brown rice, Quinoa, Barley, Bulgur, Whole grain pasta, Farro, Oats, Whole grain bread, Whole grain tortillas, Millet

Lean proteins:
Chicken breast, Turkey, Fish (salmon, tuna, mackerel, sardines), Shrimp, Tofu, Tempeh, Edamame, Greek yogurt, Cottage cheese, Eggs

Legumes:
Lentils, Chickpeas, Black beans, Kidney beans, Pinto beans, Navy beans, Cannellini beans, Peas, Lima beans, Mung beans

Nuts and seeds:
Almonds, Walnuts, Pistachios, Chia seeds, Flaxseeds, Pumpkin seeds, Sunflower seeds, Hemp seeds, Pecans, Cashews

Healthy fats:
Avocado, Olive oil, Coconut oil, Flaxseed oil, Nuts and nut butters (almond, peanut, cashew)
Seeds and seed butters (sunflower, pumpkin), Fatty fish (salmon, sardines, mackerel), Olives, Dark chocolate (70% or higher cocoa content), Greek yogurt (full-fat, unsweetened)

Beverages:
Water, Green tea, Black tea, Herbal tea, Coffee (black or with minimal added sugar/cream), Sparkling water, Lemon water, Vegetable juice (low sodium), Unsweetened almond milk, Coconut water (unsweetened)

*Remember to practice portion control, as even healthy foods can contribute to weight gain if consumed in excess.

<1500 calorie Sample meal plans for 5 days>

	DAY1	DAY2	DAY3	DAY4	DAY5
Breakfast	Smoothie: 1 cup unsweetened almond milk, 1/2 banana, 1/2 cup frozen berries, 1 scoop protein powder, 1 tablespoon flaxseed	1 slice of whole-grain toast with 1 tablespoon almond butter<br>1 medium banana<br>1 cup skim milk or unsweetened almond milk	1 cup cooked oatmeal with 1/2 cup mixed berries, 1 tablespoon chopped walnuts, and a drizzle of honey	Veggie scramble: 2 eggs, 1/4 cup chopped spinach, 1/4 cup diced bell pepper, 1/4 cup diced tomatoes, and 1/4 avocado<br>1 slice of whole-grain toast	2 slices of whole-grain toast with mashed 1/2 avocado and 1 poached egg<br>A pinch of black pepper and paprika for seasoning
Lunch	2 cups mixed greens with 1/2 cup cherry tomatoes, 1/2 avocado, 1/4 cup shredded carrots, and 3 ounces grilled chicken breast<br>1 tablespoon vinaigrette dressing<br>1 small apple	Vegetable stir-fry with 1 cup mixed veggies, 3 ounces tofu or chicken, and 1/2 cup cooked brown rice<br>1 tablespoon low-sodium soy sauce	3 ounces canned tuna mixed with 1 tablespoon light mayo, chopped celery, and diced red onion, served on a bed of lettuce<br>5 whole-grain crackers<br>1 medium orange	Turkey and avocado wrap: 1 whole-grain tortilla, 3 ounces sliced turkey, 1/4 avocado, lettuce, tomato, and mustard<br>1 cup baby carrots	Spinach and feta stuffed pita: 1 small whole-grain pita filled with 1/4 cup crumbled feta cheese, 1/2 cup baby spinach, 1/4 cup diced cucumber, 1/4 cup diced tomatoes, and a drizzle of balsamic vinaigrette
Dinner	4 ounces baked salmon<br>1/2 cup cooked quinoa<br>1 cup steamed broccoli<br>1 teaspoon olive oil and lemon juice for seasoning	4 ounces grilled chicken breast<br>1/2 cup cooked brown rice<br>1 cup steamed green beans<br>1 teaspoon olive oil and lemon juice for seasoning	4 ounces baked tilapia<br>1 small baked sweet potato with 1 teaspoon butter<br>1 cup steamed asparagus<br>1 teaspoon olive oil and lemon juice for seasoning	4 ounces lean beef steak<br>1/2 cup cooked barley<br>1 cup steamed Brussels sprouts<br>1 teaspoon olive oil and balsamic vinegar for seasoning	4 ounces baked shrimp<br>1/2 cup cooked whole grain pasta with 1/2 cup tomato sauce and sautéed spinach<br>1 teaspoon olive oil and crushed red pepper flakes for seasoning
Snack	1 cup plain Greek yogurt with 1/2 cup mixed berries<br>1/4 cup hummus with 1 cup raw vegetables (bell peppers, carrots, cucumbers)	1 medium apple with 1 tablespoon peanut butter<br>1 ounce of almonds	1/2 cup cottage cheese with 1/2 cup diced pineapple<br>1/4 cup roasted chickpeas	1 medium pear with 1 ounce of cheese<br>1 cup edamame (in the pod)	1 small apple with 1 ounce of cheddar cheese<br>1 cup raw vegetables (bell peppers, carrots, cucumbers) with 2 tablespoons Greek yogurt-based ranch dip
