# Supplementary material for: Qualitative evaluation of artificial intelligence-generated weight management diet plans
Source: Front Nutr. 2024 Mar 21;11:1374834. doi: 10.3389/fnut.2024.1374834 (PMC10991711; doi:10.3389/fnut.2024.1374834)
Supplement: Supplementary file 2 [file Data_Sheet_2.pdf]

**Personalized Diet Plan**

This is a weight loss diet plan designed for a patient who has a medical history of **stroke, chronic renal insufficiency and severe acid reflux disease**. Additionally, the patient has a **food allergy to shellfish** and has a preference for **Spanish cuisine**.

**<Introduction>**

1. Eat a balanced diet: Include a variety of fruits, vegetables, whole grains, lean proteins, and low-fat dairy products in your meals.
2. Control portion sizes: Eat smaller, more frequent meals throughout the day instead of large meals. Be mindful of serving sizes and avoid overeating.
3. Limit sodium intake: Choose low-sodium options and avoid adding extra salt to your meals. Aim for less than 1,500 mg of sodium per day, as recommended by the American Heart Association.
4. Choose heart-healthy fats: Incorporate healthy fats from sources like olive oil, avocados, and nuts, while limiting saturated and trans fats found in fried foods, processed snacks, and high-fat meats.
5. Focus on lean proteins: Select lean protein sources such as skinless poultry, fish (excluding shellfish), legumes, and tofu, while avoiding high-fat and fried proteins that can trigger GERD symptoms.
6. Increase fiber intake: Consume high-fiber foods like fruits, vegetables, whole grains, and legumes to help with weight loss, blood sugar control, and cholesterol management.
7. Avoid GERD triggers: Limit or avoid foods and beverages that can trigger GERD symptoms, including spicy foods, high-fat meals, chocolate, caffeine, and carbonated beverages.
8. Stay hydrated: Drink at least 8 cups of water daily, or as recommended by your healthcare provider, to support kidney function and promote a feeling of fullness.
9. Limit processed and sugary foods: Cut back on processed and sugary foods, such as candies, cookies, and sugary beverages, which can contribute to weight gain and provide little nutritional value.
10. Monitor and adjust: Track your progress regularly and consult with your healthcare team to make any necessary adjustments to your diet plan.

**<Food to Choose>**

**Fruits and vegetables:**
Low-potassium fruits: Apples, berries, grapes, pineapples, plums, and tangerines.
Low-potassium vegetables: Cabbage, cauliflower, cucumber, green beans, lettuce, and zucchini.
High-fiber fruits and vegetables: Broccoli, Brussels sprouts, carrots, pears, and spinach.
**Grains:**
Whole grains: Brown rice, barley, bulgur, whole wheat pasta, and whole-grain bread.
High-fiber grains: Oats, quinoa, and whole-grain cereals.
**Protein sources:**
Poultry: Skinless chicken or turkey.
Fish: Salmon, tuna, or other non-shellfish options.
Plant-based proteins: Lentils, chickpeas, black beans, kidney beans, and tofu.
Low-fat dairy: Skim milk, low-fat yogurt, and low-fat cheese.
**Healthy fats:**
Avocado.
Nuts and seeds: Almonds, walnuts, chia seeds, and flaxseeds (in moderation due to phosphorus content).
Oils: Olive oil and canola oil.
**Beverages:**
Water, herbal tea, and other non-caffeinated drinks.
Herbs and spices:
Basil, cilantro, dill, oregano, rosemary, thyme, and other herbs and spices to add flavor without adding sodium.

**<Detailed diet plan divided into meal categories>**

**Breakfast:**

1. Choose whole grains: Opt for whole-grain cereals, bread, or a Spanish-style oatmeal called "avena". This will provide fiber and help with weight loss, blood sugar control, and cholesterol management.
2. Add protein: Include a lean protein source like low-fat yogurt, queso fresco (fresh cheese), or a Spanish omelette (tortilla española) made with minimal oil or butter.
3. Incorporate fruits: Add fresh or frozen fruits to your cereal, yogurt, or oatmeal for added nutrients and fiber.

**Lunch:**

1. Focus on vegetables: Make a salad with a variety of colorful vegetables, such as leafy greens, tomatoes, cucumbers, and bell peppers. Consider a Spanish-style salad like "ensalada mixta" but with a reduced sodium content.
2. Include lean protein: Add a serving of skinless chicken, turkey, fish (excluding shellfish), legumes, or tofu to your salad for a protein boost. Try "pollo al ajillo" (garlic chicken) or "bacalao a la vizcaína" (cod in tomato sauce) as Spanish-inspired options.
3. Use heart-healthy dressings: Opt for an olive oil-based vinaigrette, like "alioli" made without garlic (to avoid GERD triggers) or low-sodium dressing instead of high-fat or creamy dressings.

**Dinner:**

1. Plan a balanced plate: Fill half of your plate with non-starchy vegetables, a quarter with whole grains (like brown rice, whole-grain pasta, or whole-wheat couscous), and a quarter with lean protein (skinless poultry, fish, legumes, or tofu).
2. Cook using heart-healthy methods: Use cooking methods like baking, grilling, or steaming instead of frying to avoid excess fat. Try Spanish-inspired dishes like "pisto" (vegetable stew) or "escabeche de pescado" (marinated fish) without shellfish.
3. Limit sodium: Choose low-sodium seasonings and sauces, and avoid adding extra salt to your meals. Modify traditional Spanish recipes by reducing the salt content.

**Snacks:**

1. Opt for healthy options: Choose snacks like fresh fruits, "pan con tomate" (bread with tomato) using whole-grain bread, or unsalted almonds.
2. Limit portion sizes: Be mindful of serving sizes to avoid overeating.

**Beverages:**

1. Drink plenty of water: Aim for at least 8 cups of water daily, or as recommended by your healthcare provider.
2. Avoid GERD triggers: Limit or avoid beverages like coffee, tea, and carbonated drinks, which can trigger GERD symptoms.

**General Tips:**

1. Read food labels: Check for sodium content, added sugars, and unhealthy fats when shopping for groceries.
2. Plan meals in advance: Create a weekly meal plan to help you make healthier choices and avoid last-minute, unhealthy options.
3. Seek support: Consult with your healthcare team or a registered dietitian for personalized recommendations based on your specific medical history and needs.

# <Sample meal plans for 5 days>

| | DAY1 | DAY2 | DAY3 | DAY4 | DAY5 |
|---|---|---|---|---|---|
| Breakfast | Spanish omelet with spinach, onions, and bell peppers; whole-grain toast; fresh fruit salad (apple, grapes, and pineapple) | Whole-grain toast with tomato and avocado; a bowl of mixed berries | Whole-grain cereal with skim milk; a bowl of fresh fruit (apple, pineapple, and berries) | Overnight oats with skim milk, chia seeds, and diced apple; herbal tea | Whole-grain toast with a thin layer of low-fat cream cheese and sliced tomato; fresh fruit salad (pineapple, grapes, and berries) |
| Lunch | Lentil and vegetable stew; mixed green salad with olive oil and lemon dressing | Chickpea and spinach stew; mixed green salad with cucumber, olives, and a balsamic vinaigrette | White bean salad with tomatoes, red onion, and parsley; whole-grain bread; mixed greens | Gazpacho with whole-grain croutons; mixed green salad with olive oil and lemon dressing | Spanish-style chicken and vegetable soup; mixed green salad with a balsamic vinaigrette |
| Dinner | Grilled chicken with Romesco sauce; sautéed green beans and zucchini; brown rice | Baked cod with roasted red peppers and onions; steamed cauliflower; quinoa | Turkey and vegetable stuffed bell peppers; barley with a side of green beans | Grilled tuna with a tomato and olive salsa; sautéed zucchini and eggplant; brown rice | Eggplant and tomato bake (Spanish moussaka) with a side of quinoa; steamed green beans |
| Snack | Low-fat yogurt with honey and a few almonds | Fresh fruit (tangerine, grapes) and a small handful of walnuts | Low-fat yogurt with cinnamon and chopped pear | Fresh fruit (plum, tangerine) and a few almonds | Low-fat yogurt with honey and a small handful of walnuts |
